# 4-Benzyl-7-chloro-2*H*-1,4-benz­oxazin-3(4*H*)-one

**DOI:** 10.1107/S1600536809012586

**Published:** 2009-04-10

**Authors:** Zhu-Bo Li, Hao Yang, Yong-Sheng Xie, Chen-Guang Zhao

**Affiliations:** a College of Pharmaceutical Sciences, Southwest University, Chongqing 400715, People’s Republic of China; b College of Horticulture and Landscape Architecture, Southwest University, Chongqing 400715, People’s Republic of China; c School of Chemistry & Chemical Engineering, Shandong University, Jinan 250100, People’s Republic of China

## Abstract

In the title compound, C_15_H_12_ClNO_2_, the two benzene rings are nearly perpendicular to each other [dihedral angle = 89.99 (13)°]. The O atom of the six-membered heterocyclic ring is disordered over two sites in a ratio of 0.46 (4):0.54 (4) and is displaced from the mean plane formed by other five atoms, resulting an envelope conformation of the six-membered hetercycle ring.

## Related literature

For the biological activity of benzo[*b*][1,4]oxazin-3(4*H*)-ones, see: Frechette & Weidner-Wells (1997 ▶); Maag *et al.* (2004 ▶). For the synthesis of benzo[*b*][1,4]oxazin-3(4*H*)-ones, see: Zuo *et al.* (2008 ▶). For a related structure, see: Cao *et al.* (2004 ▶).
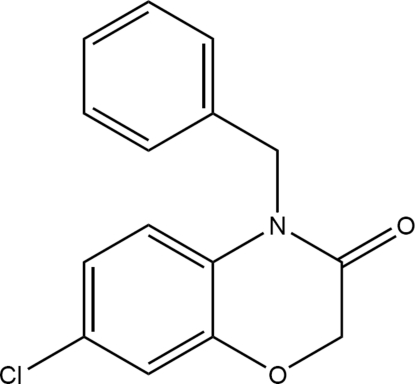


## Experimental

### 

#### Crystal data

C_15_H_12_ClNO_2_
*M* *_r_* = 273.71
Monoclinic, 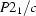

*a* = 5.0894 (11) Å
*b* = 12.630 (3) Å
*c* = 20.070 (4) Å
β = 91.833 (4)°
*V* = 1289.4 (5) Å^3^
*Z* = 4
Mo *K*α radiation
μ = 0.29 mm^−1^
*T* = 298 K
0.16 × 0.14 × 0.10 mm

#### Data collection

Bruker SMART CCD area-detector diffractometer
Absorption correction: multi-scan (*SADABS*; Bruker, 2005 ▶) *T*_min_ = 0.955, *T*_max_ = 0.971
6606 measured reflections
2268 independent reflections
1402 reflections with *I* > 2σ(*I*)
*R*_int_ = 0.031

#### Refinement

*R*[*F* ^2^ > 2σ(*F* ^2^)] = 0.044
*wR*(*F* ^2^) = 0.121
*S* = 1.02
2268 reflections
182 parameters
H-atom parameters constrained
Δρ_max_ = 0.18 e Å^−3^
Δρ_min_ = −0.21 e Å^−3^

### 

Data collection: *SMART* (Bruker 2005 ▶); cell refinement: *SAINT* (Bruker 2005 ▶); data reduction: *SAINT*; program(s) used to solve structure: *SHELXS97* (Sheldrick, 2008 ▶); program(s) used to refine structure: *SHELXL97* (Sheldrick, 2008 ▶); molecular graphics: *XP* in *SHELXTL* (Sheldrick, 2008 ▶); software used to prepare material for publication: *SHELXL97*.

## Supplementary Material

Crystal structure: contains datablocks I, New_Global_Publ_Block. DOI: 10.1107/S1600536809012586/xu2496sup1.cif

Structure factors: contains datablocks I. DOI: 10.1107/S1600536809012586/xu2496Isup2.hkl

Additional supplementary materials:  crystallographic information; 3D view; checkCIF report

## Figures and Tables

**Fig. 1.**
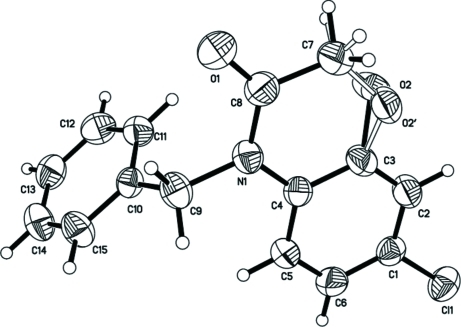
The molecular structure of the title compound, showing displacement ellipsoids drawn at the 50% probability level.

## supplementary crystallographic information

### Comment

Benzo[*b*][1,4]oxazin-3(4*H*)-ones are an important class of heteroaromatic ring systems that have shown to exhibit a wide range of biological activities such as antimicrobial (Frechette & Weidner-Wells, 1997) and selective 5-HT6 antagonistic activities (Maag *et al.*, 2004). Due to its extensive biological application, the efficient synthesis of benzo[*b*][1,4]oxazin-3(4*H*)-ones has recently received much attention (Zuo *et al.*, 2008). We report here the crystal structure of the title compound (Fig. 1). All bond lengths and angles are normal. The dihedral angle between the two benzene rings is 89.99 (13)°. The crystal packing is mainly stabilized by van der Waals interactions. In the title compound the six-membered heterocyclic ring is envelope conformation while in the related compound (Cao *et al.*, 2004) it adopts a screw-boat conformation.

### Experimental

To the solution of *N*-benzyl-2-(2,4-dichlorophenoxy)acetamide (0.620 g, 2.0 mmol) in DMF (20 ml), caesium carbonate (0.787 g, 2.4 mmol) was added. The mixture was refluxed for 2 h. After completion of the reaction (by TLC monitoring), the DMF was removed under vacuum. Water (20 ml) was added into the residue to obtain a turbid solution and it was extracted by ethyl acetate (20 ml *x* 4). The combined organic layer was washed by 1 mol/*L* of hydrochloric acid (10 ml *x* 3) and saturated sodium chloride solution (10 ml *x* 3), dried over MgSO_4_. And then the mixture was filtered and the filtrate was concentrated under reduced pressure to obtain the corresponding crude product. The product was purified by column chromatography on silica gel using ethyl acetate/hexane = 1/5 as eluent (yield 70%). Crystals suitable for X-ray diffraction were obtained by slow evaporation of a solution of the solid dissolved in ethyl acetate/hexane at room temperature for 10 days.

### Refinement

H atoms were positioned geometrically with C—H = 0.93–0.97 Å and refined in the riding-model approximation with *U*_iso_(H) = 1.2*U*_eq_(C). The O2 atom is disordered over two sites with a ratio of 0.46 (4):0.54 (4).

### Crystal data

**Table:**

C_15_H_12_ClNO_2_	*F*(000) = 568
*M**_r_* = 273.71	*D*_x_ = 1.410 Mg m^−^^3^
Monoclinic, *P*2_1_/*c*	Mo *K*α radiation, λ = 0.71073 Å
Hall symbol: -P 2ybc	Cell parameters from 1156 reflections
*a* = 5.0894 (11) Å	θ = 2.6–20.8°
*b* = 12.630 (3) Å	µ = 0.29 mm^−^^1^
*c* = 20.070 (4) Å	*T* = 298 K
β = 91.833 (4)°	Block, colorless
*V* = 1289.4 (5) Å^3^	0.16 × 0.14 × 0.10 mm
*Z* = 4	

### Data collection

**Table:**

Bruker SMART CCD area-detector diffractometer	2268 independent reflections
Radiation source: fine-focus sealed tube	1402 reflections with *I* > 2σ(*I*)
graphite	*R*_int_ = 0.031
φ and ω scans	θ_max_ = 25.1°, θ_min_ = 1.9°
Absorption correction: multi-scan (*SADABS*; Bruker, 2005)	*h* = −6→6
*T*_min_ = 0.955, *T*_max_ = 0.971	*k* = −15→10
6606 measured reflections	*l* = −23→22

### Refinement

**Table:**

Refinement on *F*^2^	Primary atom site location: structure-invariant direct methods
Least-squares matrix: full	Secondary atom site location: difference Fourier map
*R*[*F*^2^ > 2σ(*F*^2^)] = 0.044	Hydrogen site location: inferred from neighbouring sites
*wR*(*F*^2^) = 0.121	H-atom parameters constrained
*S* = 1.02	*w* = 1/[σ^2^(*F*_o_^2^) + (0.0527*P*)^2^ + 0.1942*P*] where *P* = (*F*_o_^2^ + 2*F*_c_^2^)/3
2268 reflections	(Δ/σ)_max_ < 0.001
182 parameters	Δρ_max_ = 0.18 e Å^−^^3^
0 restraints	Δρ_min_ = −0.21 e Å^−^^3^

### Special details

**Table:**

Geometry. All e.s.d.'s (except the e.s.d. in the dihedral angle between two l.s. planes) are estimated using the full covariance matrix. The cell e.s.d.'s are taken into account individually in the estimation of e.s.d.'s in distances, angles and torsion angles; correlations between e.s.d.'s in cell parameters are only used when they are defined by crystal symmetry. An approximate (isotropic) treatment of cell e.s.d.'s is used for estimating e.s.d.'s involving l.s. planes.
Refinement. Refinement of *F*^2^ against ALL reflections. The weighted *R*-factor *wR* and goodness of fit *S* are based on *F*^2^, conventional *R*-factors *R* are based on *F*, with *F* set to zero for negative *F*^2^. The threshold expression of *F*^2^ > σ(*F*^2^) is used only for calculating *R*-factors(gt) *etc*. and is not relevant to the choice of reflections for refinement. *R*-factors based on *F*^2^ are statistically about twice as large as those based on *F*, and *R*- factors based on ALL data will be even larger.

### Fractional atomic coordinates and isotropic or equivalent isotropic displacement parameters (Å^2^)

**Table:**

	*x*	*y*	*z*	*U*_iso_*/*U*_eq_	Occ. (<1)
Cl1	1.04449 (14)	0.70775 (6)	0.03801 (4)	0.0845 (3)	
O1	−0.0247 (4)	0.38796 (16)	0.23334 (10)	0.0903 (6)	
O2	0.459 (4)	0.4090 (11)	0.1102 (14)	0.079 (4)	0.46 (4)
O2'	0.358 (5)	0.4386 (16)	0.0928 (5)	0.085 (4)	0.54 (4)
N1	0.2417 (4)	0.52888 (17)	0.21372 (9)	0.0620 (5)	
C1	0.8090 (5)	0.6568 (2)	0.09015 (12)	0.0620 (7)	
C2	0.6971 (5)	0.5605 (2)	0.07550 (13)	0.0723 (7)	
H2	0.7454	0.5233	0.0378	0.087*	
C3	0.5134 (5)	0.5196 (2)	0.11685 (13)	0.0679 (7)	
C4	0.4348 (5)	0.57338 (19)	0.17315 (11)	0.0567 (6)	
C5	0.5528 (6)	0.6694 (2)	0.18658 (13)	0.0715 (8)	
H5	0.5058	0.7070	0.2242	0.086*	
C6	0.7390 (6)	0.7116 (2)	0.14561 (14)	0.0765 (8)	
H6	0.8161	0.7767	0.1556	0.092*	
C7	0.2299 (8)	0.3790 (2)	0.13820 (16)	0.0967 (10)	
H7A	0.2446	0.3040	0.1481	0.116*	0.46 (4)
H7B	0.0913	0.3865	0.1042	0.116*	0.46 (4)
H7'A	0.3253	0.3162	0.1528	0.116*	0.54 (4)
H7'B	0.0757	0.3544	0.1131	0.116*	0.54 (4)
C8	0.1376 (6)	0.4322 (2)	0.19949 (14)	0.0713 (7)	
C9	0.1563 (5)	0.5849 (2)	0.27351 (12)	0.0673 (7)	
H9A	−0.0118	0.5564	0.2862	0.081*	
H9B	0.1306	0.6591	0.2626	0.081*	
C10	0.3484 (4)	0.57652 (19)	0.33223 (11)	0.0534 (6)	
C11	0.5208 (5)	0.4939 (2)	0.34046 (13)	0.0684 (7)	
H11	0.5248	0.4407	0.3085	0.082*	
C12	0.6897 (6)	0.4889 (2)	0.39610 (16)	0.0849 (9)	
H12	0.8071	0.4328	0.4010	0.102*	
C13	0.6842 (6)	0.5657 (3)	0.44339 (14)	0.0797 (8)	
H13	0.7962	0.5616	0.4808	0.096*	
C14	0.5167 (6)	0.6478 (3)	0.43615 (14)	0.0819 (9)	
H14	0.5142	0.7007	0.4683	0.098*	
C15	0.3484 (5)	0.6531 (2)	0.38073 (14)	0.0748 (8)	
H15	0.2327	0.7098	0.3763	0.090*	

### Atomic displacement parameters (Å^2^)

**Table:**

	*U*^11^	*U*^22^	*U*^33^	*U*^12^	*U*^13^	*U*^23^
Cl1	0.0817 (5)	0.0935 (6)	0.0780 (5)	−0.0153 (4)	0.0008 (4)	0.0149 (4)
O1	0.0999 (15)	0.0831 (13)	0.0889 (14)	−0.0188 (12)	0.0197 (12)	0.0042 (11)
O2	0.081 (6)	0.055 (5)	0.103 (8)	−0.012 (4)	0.024 (6)	−0.024 (5)
O2'	0.111 (9)	0.085 (6)	0.062 (3)	−0.030 (6)	0.014 (4)	−0.019 (3)
N1	0.0640 (13)	0.0662 (13)	0.0556 (12)	−0.0020 (11)	−0.0015 (10)	−0.0049 (10)
C1	0.0630 (16)	0.0635 (16)	0.0585 (15)	−0.0031 (13)	−0.0109 (13)	0.0075 (13)
C2	0.0794 (18)	0.0759 (19)	0.0618 (16)	−0.0103 (15)	0.0068 (14)	−0.0117 (14)
C3	0.0764 (18)	0.0629 (16)	0.0642 (16)	−0.0124 (14)	−0.0004 (14)	−0.0120 (14)
C4	0.0628 (15)	0.0569 (15)	0.0497 (14)	−0.0002 (13)	−0.0074 (12)	0.0003 (12)
C5	0.098 (2)	0.0594 (16)	0.0570 (15)	−0.0021 (15)	0.0018 (15)	−0.0067 (13)
C6	0.099 (2)	0.0597 (16)	0.0709 (18)	−0.0122 (16)	−0.0035 (16)	0.0018 (15)
C7	0.135 (3)	0.0709 (19)	0.085 (2)	−0.024 (2)	0.028 (2)	−0.0136 (18)
C8	0.0770 (19)	0.0689 (18)	0.0678 (17)	−0.0049 (16)	−0.0007 (15)	0.0044 (15)
C9	0.0586 (15)	0.0778 (18)	0.0653 (16)	0.0050 (13)	0.0013 (13)	−0.0107 (14)
C10	0.0501 (14)	0.0603 (15)	0.0501 (13)	0.0006 (12)	0.0070 (11)	0.0018 (12)
C11	0.0787 (18)	0.0624 (16)	0.0640 (17)	0.0060 (14)	0.0017 (14)	0.0003 (13)
C12	0.094 (2)	0.075 (2)	0.085 (2)	0.0196 (16)	−0.0067 (18)	0.0158 (17)
C13	0.086 (2)	0.094 (2)	0.0594 (17)	−0.0009 (18)	−0.0062 (14)	0.0101 (17)
C14	0.083 (2)	0.097 (2)	0.0655 (18)	−0.0023 (18)	−0.0002 (16)	−0.0212 (17)
C15	0.0673 (17)	0.0811 (19)	0.0758 (18)	0.0175 (14)	0.0001 (15)	−0.0147 (16)

### Geometric parameters (Å, °)

**Table:**

Cl1—C1	1.739 (3)	C7—H7A	0.9700
O1—C8	1.222 (3)	C7—H7B	0.9700
O2—C7	1.363 (11)	C7—H7'A	0.9700
O2—C3	1.429 (11)	C7—H7'B	0.9700
O2'—C7	1.363 (9)	C9—C10	1.511 (3)
O2'—C3	1.372 (9)	C9—H9A	0.9700
N1—C8	1.357 (3)	C9—H9B	0.9700
N1—C4	1.413 (3)	C10—C11	1.370 (3)
N1—C9	1.471 (3)	C10—C15	1.372 (3)
C1—C6	1.368 (4)	C11—C12	1.389 (4)
C1—C2	1.370 (3)	C11—H11	0.9300
C2—C3	1.371 (3)	C12—C13	1.357 (4)
C2—H2	0.9300	C12—H12	0.9300
C3—C4	1.388 (3)	C13—C14	1.348 (4)
C4—C5	1.376 (3)	C13—H13	0.9300
C5—C6	1.381 (4)	C14—C15	1.384 (4)
C5—H5	0.9300	C14—H14	0.9300
C6—H6	0.9300	C15—H15	0.9300
C7—C8	1.491 (4)		
			
C7—O2—C3	113.6 (7)	O2'—C7—H7'A	114.1
C7—O2'—C3	117.4 (6)	C8—C7—H7'A	106.6
C8—N1—C4	120.7 (2)	H7B—C7—H7'A	129.7
C8—N1—C9	118.7 (2)	O2—C7—H7'B	124.2
C4—N1—C9	120.5 (2)	O2'—C7—H7'B	103.0
C6—C1—C2	120.4 (3)	C8—C7—H7'B	107.6
C6—C1—Cl1	120.3 (2)	H7A—C7—H7'B	81.4
C2—C1—Cl1	119.3 (2)	H7'A—C7—H7'B	106.6
C1—C2—C3	119.4 (2)	O1—C8—N1	124.1 (3)
C1—C2—H2	120.3	O1—C8—C7	119.3 (3)
C3—C2—H2	120.3	N1—C8—C7	116.6 (3)
C2—C3—O2'	117.7 (5)	N1—C9—C10	113.70 (19)
C2—C3—C4	121.9 (2)	N1—C9—H9A	108.8
O2'—C3—C4	118.2 (8)	C10—C9—H9A	108.8
C2—C3—O2	116.5 (5)	N1—C9—H9B	108.8
C4—C3—O2	119.5 (8)	C10—C9—H9B	108.8
C5—C4—C3	117.1 (2)	H9A—C9—H9B	107.7
C5—C4—N1	123.0 (2)	C11—C10—C15	117.8 (2)
C3—C4—N1	119.9 (2)	C11—C10—C9	122.9 (2)
C4—C5—C6	121.8 (3)	C15—C10—C9	119.3 (2)
C4—C5—H5	119.1	C10—C11—C12	120.6 (3)
C6—C5—H5	119.1	C10—C11—H11	119.7
C1—C6—C5	119.4 (3)	C12—C11—H11	119.7
C1—C6—H6	120.3	C13—C12—C11	120.2 (3)
C5—C6—H6	120.3	C13—C12—H12	119.9
O2—C7—C8	120.6 (7)	C11—C12—H12	119.9
O2'—C7—C8	118.1 (8)	C14—C13—C12	120.1 (3)
O2—C7—H7A	107.2	C14—C13—H13	120.0
O2'—C7—H7A	129.9	C12—C13—H13	120.0
C8—C7—H7A	107.2	C13—C14—C15	119.8 (3)
O2—C7—H7B	107.2	C13—C14—H14	120.1
O2'—C7—H7B	80.1	C15—C14—H14	120.1
C8—C7—H7B	107.2	C10—C15—C14	121.5 (3)
H7A—C7—H7B	106.8	C10—C15—H15	119.3
O2—C7—H7'A	85.8	C14—C15—H15	119.3
			
C6—C1—C2—C3	0.2 (4)	C4—C5—C6—C1	0.0 (4)
Cl1—C1—C2—C3	179.3 (2)	C3—O2—C7—O2'	60.5 (13)
C1—C2—C3—O2'	163.7 (15)	C3—O2—C7—C8	−34 (3)
C1—C2—C3—C4	0.6 (4)	C3—O2'—C7—O2	−69.5 (14)
C1—C2—C3—O2	−163.1 (15)	C3—O2'—C7—C8	34 (3)
C7—O2'—C3—C2	162.2 (16)	C4—N1—C8—O1	178.0 (2)
C7—O2'—C3—C4	−34 (3)	C9—N1—C8—O1	0.1 (4)
C7—O2'—C3—O2	66.7 (14)	C4—N1—C8—C7	−2.3 (4)
C7—O2—C3—C2	−162.9 (15)	C9—N1—C8—C7	179.8 (2)
C7—O2—C3—O2'	−62.9 (13)	O2—C7—C8—O1	−160.9 (16)
C7—O2—C3—C4	33 (3)	O2'—C7—C8—O1	164.1 (14)
C2—C3—C4—C5	−1.1 (4)	O2—C7—C8—N1	19.4 (17)
O2'—C3—C4—C5	−164.0 (13)	O2'—C7—C8—N1	−15.6 (15)
O2—C3—C4—C5	162.1 (14)	C8—N1—C9—C10	100.5 (3)
C2—C3—C4—N1	179.0 (2)	C4—N1—C9—C10	−77.4 (3)
O2'—C3—C4—N1	16.0 (14)	N1—C9—C10—C11	−25.9 (3)
O2—C3—C4—N1	−17.9 (14)	N1—C9—C10—C15	155.7 (2)
C8—N1—C4—C5	−177.8 (2)	C15—C10—C11—C12	−0.3 (4)
C9—N1—C4—C5	0.1 (3)	C9—C10—C11—C12	−178.7 (3)
C8—N1—C4—C3	2.1 (3)	C10—C11—C12—C13	0.6 (4)
C9—N1—C4—C3	−180.0 (2)	C11—C12—C13—C14	−0.8 (5)
C3—C4—C5—C6	0.8 (4)	C12—C13—C14—C15	0.7 (5)
N1—C4—C5—C6	−179.3 (2)	C11—C10—C15—C14	0.2 (4)
C2—C1—C6—C5	−0.5 (4)	C9—C10—C15—C14	178.6 (2)
Cl1—C1—C6—C5	−179.6 (2)	C13—C14—C15—C10	−0.4 (4)
